# Capturing Multiple Disease Resistance in Wheat through Intergeneric Hybridization

**DOI:** 10.3390/biology10070631

**Published:** 2021-07-08

**Authors:** George Fedak, Dawn Chi, Colin Hiebert, Tom Fetch, Brent McCallum, Allen Xue, Wenguang Cao

**Affiliations:** 1 Ottawa Research and Development Centre, Agriculture and Agri-Food Canada, Ottawa, ON K1A 0C6, Canada; dawn.chi@agr.gc.ca (D.C.); allen.xue@agr.gc.ca (A.X.); wenguang.cao@agr.gc.ca (W.C.); 2 Morden Research and Development Centre, Agriculture and Agri-Food Canada, Morden, MB R6M 1Y5, Canada; colin.hiebert@agr.gc.ca (C.H.); tom.fetch@agr.gc.ca (T.F.); brent.McCallum@agr.gc.ca (B.M.)

**Keywords:** intergeneric hybrids, wheat, disease resistance, Fusarium, leaf rust, stem rust, powdery mildew

## Abstract

**Simple Summary:**

Providing disease resistance in our crop plants is our ongoing exercise for plant pathologists/breeders/geneticists. Pathogens are continually evolving and releasing new variants. The variants arise through mutations or through sexual cycles on their respective alternate hosts. Thus, the search continues for unique genes for resistance. Another newer concept is the “pyramiding” of resistance genes. It has been shown that a cultivar may last only 3 years or so before being overcome by a new variant of the pathogen. The release of new cultivars with up to four resistance genes will delay their breakdown. In our ongoing work we have also produced pyramids containing a combination of resistance genes, including *SrCad* for resistance to new races of stem rust, *Lr34*, which is a major gene for resistance to leaf rust, and *Fhb1,* which is a common FHB QTL. This required the production of a series of doubled haploids (DH) to produce lines containing all four genes in reasonable-sized populations. A complex series of four-way crosses were required to generate the various gene combinations. In the studies reported here, the essential tools for marker-assisted-selection are produced, i.e., mapping populations containing the resistance genes and molecular makers assigned to each gene. It should be possible to simultaneously manipulate several resistance genes from existing genetic stocks without requiring complex cross combinations.

**Abstract:**

Derivatives from 4 species from the secondary gene pool of wheat—1 diploid (*T. monococcum*), 2 tetraploid (*T. carthlicum*; *T. timopheevi*), and 1 hexaploid (*T. miguschovae*)—were screened for resistance to Fusarium head blight, leaf rust, stem rust, and stripe rust. Where screening, genetic studies, and mapping were completed it was shown that all species carried resistance to multiple plant diseases. Some derived lines carried resistance to up to four different diseases. Where mapping was completed, it was shown that different diseases mapped to different chromosomes within any one accession.

## 1. Introduction

The most important diseases of wheat (*Triticum aestivum*) in all temperate wheat-growing regions of the world are the rusts caused by *Puccinia triticina* (leaf rust), *P. striiformis* (stripe rust), and *P. graminis* (stem rust). Fusarium head blight (FHB), caused by *Fusarium graminearum,* has become a serious disease. It not only causes yield reductions but also deposits a vomitoxin in the seed that is toxic to humans and other animals. Other common wheat diseases with a significant commercial impact on wheat production are powdery mildew (*Blumeria graminis f.* sp. *Tritici*) and loose smut (*Ustilags tritici*). 

All these diseases are constantly mutating and recombining to create new races of each species of pathogen [1]. This requires an endless supply of new resistance genes for each disease. There is insufficient variability in the primary gene pool to keep up with the demand. There is abundant variability in the secondary and tertiary gene pool for all diseases. We have concentrated our efforts on recognizing variability for resistance to Fusarium head blight and introgressing the resistance into wheat.

## 2. Materials and Methods

### 2.1. Plant Materials

The origins of the 4 genotypes reported in this study are listed in Table 1, and procedures for introgression are detailed in the narrative.

#### 2.1.1. Blackbird

Initial screening revealed type-II resistance to FHB. A DH mapping population of 91 lines was produced through wheat × maize pollination from an F1 hybrid between Blackbird and Strongfield.

#### 2.1.2. M321

Initially, 260 accessions of *T. monococcum* obtained from M. Trottet of the Institut national de la recherché agronomique (INRA) were screened for FHB resistance for type-I resistance. A second cycle of screening identified FHB resistance in line 10-1. This was crossed and backcrossed once to the AC domain with concurrent FHB screening to produce M321. A DH mapping population of 120 lines was produced from the F1 hybrid of M321 × Superb. That population has been phenotyped for FHB resistance and leaf rust. In addition, to date M321 has been evaluated for resistance to powdery mildew and stem rust.

#### 2.1.3. TC67

Accession PI 343447 of *T. timopheevi* was crossed to Crocus and 535 progeny advanced to BC1F7 by single-seed descent. One hundred progeny were selected from this population and tested for resistance to FHB. This resulted in the selection of TC67 that showed a degree of resistance [8]. TC67 was crossed to AC Brio to produce a mapping population of 230 lines.

#### 2.1.4. MSB55-1

*T. miguschovae* is an amphiploid produced by the crossing of *T. militinae* (genome AG) with *Ae. squarrosa* (DD) [9]. In our own screening we found that it also carried a degree of resistance to FHB. The amphiploid was crossed to Superb, with progeny advanced to BC2F5 from which line MSB55-1 was selected. MSB55-1 was crossed to Superb and a mapping population of 137 doubled haploid lines was produced.

### 2.2. Inoculation Methods

For type-II FHB evaluation, entire mapping populations were screened in a growth chamber. Central florets of spikes at 50% anthesis were inoculated with 10 uL of a 50,000 spores/ml suspension of 3 virulent isolates of *F. graminearum*. Plants with inoculated spikes were placed in a mist chamber at 100% relative humidity (RH) for 48 h then returned to normal growth chamber conditions of 16 h of light and day/night temperature regimes of 20/15 °C. At 21 days after inoculation, the symptoms were scored and expressed as percent infected florets. For type- I FHB resistance, mapping populations were grown in replicated field trails. At the tillering stage, corn spawn impregnated with *F. graminearum* spores were spread between the rows at the rate of 50 g per plot. At 21 days after flowering, each plot was assessed visually for incidence and severity of the disease. The 2 readings were used to calculate an FHB index that was used in resistance calculations.

For stem rust assays, the mapping populations plus check varieties were evaluated with stem rust race TTKSK. Here 10 plants of each DH progeny were assessed for seedling resistance at 14 days post-inoculation using a 0–4 scale. Infection types from 0 to 2+ were considered resistant responses, whereas those with scores of 3–4 were considered to be susceptible responses [10].

For leaf rust resistance analysis, the DH lines and check varieties were grown as 2 sets of lines with 3 seeds per line in flats in a greenhouse. They were inoculated with urediniospore suspensions in an oil carrier with isolates 12-3 MBDS and 06-1-1 TDBG [1,11]. The inoculated flats were misted for 12 h and symptoms recorded at 14 days after inoculation. Ratings of 0, 1, and 2 indicated a resistant reaction, whereas 3 and 3+ indicated susceptible reactions [10]. 

## 3. Results

### 3.1. Blackbird

The mapping population derived from Blackbird × Strongfield was phenotyped by point inoculation to detect type-II FHB resistance. Resistance QTL were mapped on chromosomes 2B and 6B [3] (Table 1). The latter was the same QTL as detected previously in hexaploid wheat, whereas the 2B QTL is unique. The same population was analyzed for type-I resistance and QTL mapped on chromosome 1D [2]. In more detailed subsequent studies, using the 90K Infinium Select Chip as a marker system, FHB resistance QTL were detected on chromosomes 1A, 2A, 3A, and 6B, with the one on chromosome 1A being most consistent over environments [4]. Loose smut resistance was mapped to chromosome 6B of Blackbird [5].

### 3.2. M321

The DH mapping population derived from M321 × Superb was phenotyped three times for FHB, and after SSR mapping, a QTL was located on chromosome 5A linked to marker *Xwmc 705* [12]. Subsequent mapping with SNP markers [Boyle et al., unpublished] revealed FHB-resistant QTL on chromosome 5A and leaf rust-resistant QTL on chromosome 1B. The leaf rust resistance gene on chromosome 1B was located in a similar region to the stem rust gene *Sr*71; however, further testing is required to verify if they are the same genes.

### 3.3. TC67

The TC67 × Brio mapping population was screened extensively for type-I and type-II FHB resistance, for Fusarium-damaged kernels, and for deoxynivalenol (DON) content. As a result of this analysis, a major QTL was mapped on chromosome 5A that covered type-I and type-II resistance [6]. A number of other minor QTL were detected for the other traits, but since they were not significant, they were not reported. 

Preliminary data indicate that TC67 also has resistance to stem rust and stripe rust. QTL mapping needs to be performed to identify the chromosome locations of these resistances.

### 3.4. MSB55-1 

*T. miguschovae,* soon after its synthesis, was found to carry resistance to leaf rust which was subsequently mapped to chromosomes 7B and 1D [7]. The 7D resistance may have originated in *Ae. squarossa* and the 1D QTL may have come from *T. militinae*. It also has been reported to have resistance to stem rust, stripe rust, and mildew. 

In our studies, the mapping population was screened for type-II FHB resistance and with the aid of SNP markers, QTL were mapped on chromosomes 2D and 5A.

## 4. Discussion

This study shows that of the four genotypes studied, all had resistance to four to five different diseases. In most cases the resistance factors mapped to different chromosomes. Studies are continuing to develop markers for all the sources of resistance shown in Table 1.

Multiple disease resistance is a fairly common phenomenon and often can be traced to a single chromosome or chromosome translocation, especially when dealing with progeny from interspecific or intergeneric hybrids [13], For example an intact chromosome 6Ai #2 from *Th. intermedium* contributed resistance to leaf rust and powdery mildew and moderate resistance to stem rust and yellow rust when substituted for chromosome 6D of bread wheat [14]. 

The 1B/1R translocation [15], a translocation involving the short arm of rye chromosome 1R translocated to the long arm of chromosome 1B of wheat, carries resistance to multiple diseases, enhances yield and yield components [16], and presents unique quality components. It has been disseminated worldwide, at times inadvertently. For example, 50% of the breeding lines at CIMMYT contained that translocation because of its enhanced attributes. 

Mapping populations have been produced for all sources of resistance. QTL analysis needs to be completed for some combinations. Studies to date indicate these are unique genes. They have not as yet been deployed into commercial cultivars, but initial hybridizations have been completed to initiate the process. 

The key component to marker-assisted selection (MAS) is the mapping of markers in close proximity to resistance genes in question. The marker information can be used to “pyramid” multiple genes for resistance to a single pathogen or genes for resistance to multiple pathogens. In our ongoing studies both approaches have been used. Using a combination of DH technology and molecular markers applied to complex F1 hybrids, pyramids were produced that contained Ug99-resistance genes *SrCad* and *Sr33*, plus *Lr34*, *Fhb1*, and *Bt10* linked to *SrCad* [17]. Progeny were produced with any combination of any number of those five genes. To manipulate those five genes in progeny of complex crosses would have been virtually impossible through the use of conventional F2 populations. In this case, 80 DH lines were produced from 4-way crosses using the wheat × maize pollination method. 

By means of MAS and DH technology, five stem rust resistance genes were used to produce pyramids with combinations of up to four stem rust resistant genes [18]. A total of 13 pyramids with 2 to 4 resistance genes were produced.

Multiple disease resistance is evolving and with more intensive screening, more examples are being described that carry resistance to multiple diseases.

For example, among the numerous translocations observed in the progeny of Crocus × *E. repens* hybrids, we found FHB resistance was localized to wheat chromosome 3DL [19]. After more intensive screening, progenies were found that contained resistance to stripe rust [20].

We produced a total of 30 synthetic hexaploids employing a wide variety of *T. turgidum* and *T. tauschii* parents. The 15 tetraploid parents were *T. dicoccon*, *T. durum*, and *T. dicoccoides* accessions obtained from diverse sources. The 11 *T. tauschii* var. *strangulate* accessions were obtained from the Weizmann Research Institute in Rehovot Israel. Other disease combinations in this collection of synthetic hexaploids were: 2 lines with resistance to stripe rust and leaf rust, 2 with resistance to stem rust and leaf rust, and 4 with resistance to stripe rust and stem rust. Resistance to stem rust, leaf rust, stripe rust, and powdery mildew was detected in the various genotypes, with two of them showing resistance to all four diseases. Another example from our laboratory is that of two combinations of partial amphiploids involving *T. turgidum* and *Th. intermedium* (2n = 42, genome formula AABBEE) that had resistance to leaf rust, stem rust, and Fusarium head blight [21]. 

The above are examples taken from studies in our laboratories. The same phenomenon has been observed in other laboratories around the world. 

These observations extend the principle that the secondary and tertiary gene pools of wheat are excellent sources of variability for most diseases. In our studies we usually focus on one disease at a time. The lessons from the above examples are that screening for multiple diseases should be carried out. The discovery of multiple disease resistance in a single population should make the molecular mapping process more efficient.

## 5. Conclusions

This study provides additional examples of multiple disease resistance detected in derivatives of wide crosses with wheat. Some of the lessons learned from this study are that the secondary and tertiary gene pools of wheat are excellent reservoirs for variability for disease resistance. In some cases, they are the only sources of variability. Mapping populations are in place for the variants detected so far. Any new diseases or variants can thus be mapped directly. Newer emerging technologies can be applied to existing mapping populations to identify unique genes. 

## Figures and Tables

**Table 1:** Multiple disease resistance in derivatives from wide crosses.

Resistant Genotype	Origin (Genome)	Chromosome Location of Disease Resistance Genes/QTL							Reference
		Fusarium Head Blight		Rust			Powdery Mildew	Loose Smut	
		Type I	Type II	Leaf	Stripe	Stem			
“Blackbird”	*T. carthlicum* (AABB)	1A	-	-	-	-	-	-	[2]
		-	2B, 6B	-	-	-	-	-	[3]
		-	-	1A, 2A, 3A, 6B	-	-	-	-	[4]
		-	-	-	-	-	-	6B	[5]
TC67	*T. timopheevi* (AAGG)	5A	5A	-	-	-	-	-	[6]
		-	-	-	1B, 5D, 6B	3B, 5A	-	-	Unpublished
M321	*T. monococcum* (AA)		5A	1B, 5D, 6B	-	-	-	-	Boyle et al., (pc)
			-	-	-	√	√	-	√ Unpublished
MSB55-1	*T. miguchovae* (AAGGDD)	2D, 5A	-	-	-	-	-	-	Unpublished (FHB)
		-	-	7B, 1D	-	-	-	-	[7]
		-	-	-	√	√	√	-	√ Unpublished

## Data Availability

Not applicable.
